# Multi-Sensor Data Integration Using Deep Learning for Characterization of Defects in Steel Elements †

**DOI:** 10.3390/s18010292

**Published:** 2018-01-19

**Authors:** Grzegorz Psuj

**Affiliations:** Department of Electrical and Computer Engineering, Faculty of Electrical Engineering, West Pomeranian University of Technology, Szczecin, al. Piastow 17, Szczecin 70-310, Poland; gpsuj@zut.edu.pl; Tel.: +48-91-449-4727

**Keywords:** magnetic nondestructive testing, matrix transducer, multi-sensor data integration, large data processing, data aggregation, deep learning, convolutional neural network

## Abstract

Nowadays, there is a strong demand for inspection systems integrating both high sensitivity under various testing conditions and advanced processing allowing automatic identification of the examined object state and detection of threats. This paper presents the possibility of utilization of a magnetic multi-sensor matrix transducer for characterization of defected areas in steel elements and a deep learning based algorithm for integration of data and final identification of the object state. The transducer allows sensing of a magnetic vector in a single location in different directions. Thus, it enables detecting and characterizing any material changes that affect magnetic properties regardless of their orientation in reference to the scanning direction. To assess the general application capability of the system, steel elements with rectangular-shaped artificial defects were used. First, a database was constructed considering numerical and measurements results. A finite element method was used to run a simulation process and provide transducer signal patterns for different defect arrangements. Next, the algorithm integrating responses of the transducer collected in a single position was applied, and a convolutional neural network was used for implementation of the material state evaluation model. Then, validation of the obtained model was carried out. In this paper, the procedure for updating the evaluated local state, referring to the neighboring area results, is presented. Finally, the results and future perspective are discussed.

## 1. Introduction

One of the main features of currently built inspection systems are a short inspection time and a possibility of detailed local investigation. Therefore, it is necessary to develop both global and local methods [1,2,3]. The global methods do not require multipoint observation, which affects the time of the inspection. The evaluation of the whole structure is rapid, however, simultaneously, results of the inspection may also lead to generalization of a state or incomplete data. In consequence, this may lead to a greater risk of false evaluation of the current stage of the material. This affects the need for methods that operate on smaller areas, enabling observation and detection of even small local changes (e.g., few mm) in structure [4,5]. Taking into consideration the fact that most of the current structures are made of conductive and magnetic materials (and changes in the structure during its lifetime cause changes in electrical and magnetic properties [1,4]), the utilization of electromagnetic testing methods becomes a natural solution. It is well known that by observation of vector magnetic properties it is possible to assess various aspects of conditions of an examined structure [4,6,7,8,9,10,11,12,13,14,15]. Therefore, this paper focuses on presenting the results of work on a local electromagnetic system for nondestructive (NDT) inspection of steel elements.

Due to the need to increase reliability, acquisition speed, or completeness of information, today's nondestructive inspection systems should operate simultaneously within testing and evaluation regimes [1,4,6,7,8]. In addition to the rising requirements, they must present a wide range of sensitivity. This forces the design and construction of complex multidirectional and multi-sensor transducers [9,10,11,12,13,14,15]. The growth in the number of information sources, on the one hand, allows for an increase in the reliability and efficiency of measurement systems, on the other hand, it requires the use of sophisticated data processing and aggregation algorithms. The big data sets that must be analyzed by the systems result in a strong need of implementation of new processing algorithms. The procedures should be responsible, at the same time, for data collection, transformation, pattern search, and decision making [12,16,17,18,19,20,21]. An example of such an approach is the use of machine learning algorithms to automatically search even very complex relations between a large amount of input data [17,18,20,21,22,23]. However, the classical machine learning techniques require the design of learning features that represent the classes’ prior running of the procedure [17,20,21,22,24]. Extraction of features suitable for the classification of many real problems is one of the major tasks and, in consequence, has significant influence on the final accuracy of the utilized algorithm. The rapid development of technological solutions, operating in remote and autonomic modes, requires enormous effort to be undertaken to develop new automated learning algorithms to find hidden relationships in the analyzed problems. In recent years, there has been a steady increase in the number of successful applications of deep learning techniques, which not only carry out the classification, but also automate the procedure of designing features [16,25,26,27,28,29,30,31,32,33,34]. In general, deep architecture consists of a structure with many hidden layers leading to hierarchical feature extraction and, thus, eliminating the problem of inadequate representation of learning features. Construction of broad and extensive nets of connections between layers results in multiple possibilities of representation of the same data, focusing on different attributes [20,24,25]. In addition, one of the most important advantages of the deep architectures is the capability of creating hierarchical representations ranging from low to high levels of description. The high abstraction level of data leads to robustness of local input changes (such as location of object) and consequently to better classification performance [20,26,30,31,32,33]. There have been several deep architectures successfully utilized in recent years [25,26] such as restricted Boltzmann machine (RBM), deep belief network (DBM), autoencoder (AE), and deep convolutional neural network (DCNN) [18,20,22,23,28,30,31,32,33,34]. They were used in many problems involving speech recognition [29], natural language processing, image analysis [23,26], and computer vision [18,20,22,30,31,32,33,34]. The number of applications of deep neural networks in the field of nondestructive examination is also rapidly growing. The DCNN finds its successful utilization in many imaging inspection systems based on visual and X-ray techniques. The DCNNs were effectively introduced to detect damage in civil architecture such as concrete and steel constructions [18,20,30,31], diagnose rail infrastructure faults and examine defects [18,32,33,34], control steel quality during the production process [20,22], and improve computer tomographic reconstructions [23]. The dynamic progress is especially visible in sectors such as civil engineering or rail transportation, where there are large scale inspection problems. In those applications, the wide range of continuous quality control requires fast and automatic selection of faulty areas for further detailed assessment of local condition. In [30], the authors developed a vision-based method using DCNN to build a classifier for detection of concrete cracks, which is less sensitive to diverse imaging conditions. Another vision methodology was proposed in [31]. The authors utilized faster region-based convolutional neural network (Faster R-CNN) for quasi real-time detection of multiple damage types, such as concrete cracks, steel and bolt corrosions, or steel delamination. The proposed structures reached accuracy of around 98% in the first case and cumulatively close to 90% in the second one. Recently, a series of papers on effective automatic visual inspection of a wide spectrum of rail infrastructure was also published. Another Faster R-CNN structure operating with feature extraction, Markov Random Fields image segmentation and statistics-based texture description, was successively applied for rapid inspection time of isoelectric lines [32]. In [18], three DCNN structures of different sizes were designed for automatic detection of five types of rail track surface defects, while in [34], a methodology for failure risk assessment fed with deep network classification and visual length estimation results of squat defects was introduced. The on-line vision inspection system for power line element diagnostics was presented in [32,33]. In the first case, the cascade system incorporating two detectors (single-shot multibox SSD, and you-look-only-once YOLO detector) with DCNN structure for realizing coarse-to-fine approach for fastener defect detection was shown and discussed. The dominative utilization area of deep neural network methodology in visual and X-ray inspection systems is clearly visible. However, considering the properties of DCNN, the architecture can be effectively implemented in various NDT methods for a wide spectrum of problems requiring processing of two-dimensional data. 

The objective of this paper is to adopt and evaluate a method to carry out the interpretation of complicated responses of a matrix multi-sensor transducer that will identify different material states without definition of the human-based learning features or rules. Taking into consideration the above details, in this paper, the possibility of DCNN utilization was analyzed. The axial-symmetric transducer presents similar sensitivity for any flaws (or in general any nonuniformity) affecting the changes of the examined materials’ magnetic properties, regardless their orientation in reference to the x-y axis. Previously, the details of the construction of the utilized transducer and the preliminary results of defect detection and evaluation were presented [12,13]. The magnetic field distribution sensed by the sensor matrix depends on a location of the defected area in the material with regard to the transducer’s circumference and differs with position [12]. Therefore, a unique reconstruction of field distribution is achieved for each single position of the transducer over the material with defected area. Taking this into consideration, in this paper, utilization of the DCNN to characterize defects using the transducer’s data sensed in a single location over the steel plate was analyzed. To design the evaluation procedure and, finally, assess the system’s performance, the experiments were carried out for rectangular-shaped artificial defects. Further, in order to obtain natural patterns of field distributions dependent only on given defect arrangement, a database was constructed using finite element methods (FEM) computation results. Following the rapid development of technology and computational power, utilization of numerical analysis to increase the efficiency of measuring systems and the reliability of the inspection methods is significantly growing [10,13,35]. The numerical modeling in industrial applications is a powerful tool. It can provide a wide range of possible cases, required in calibration procedure or for purposes of referencing, without the need to conduct time-consuming and expensive measuring experiments. The performance of the carried-out numerical-simulation was assessed by comparison of the simulated and measured data. Then, the multi-label classification model was designed, trained, and validated. Next, in order to achieve higher accuracy of the classification process, the algorithm allowing for the combination of evaluation results from neighboring positions was introduced. Finally, in this paper, the results are discussed and conclusions presented.

## 2. Matrix Multi-Sensor Transducer 

**Figure 1:**
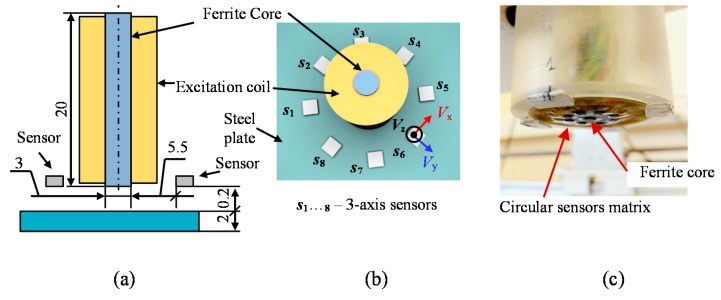
Model and photo of the multi-sensor transducer: (**a**) cross-section; (**b**) 3D view; (**c**) photo of the bottom-side, all dimensions are in [mm].

**Figure 2:**
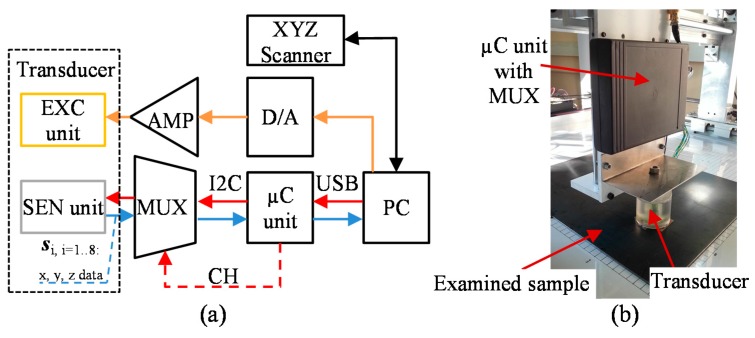
The utilized measuring system configuration diagram (**a**) and photo (**b**). EXC—excitation section; SEN—sensors; AMP—amplifier; MUX—multiplexer; CH—channel; XYZ Scanner—Cartesian coordinate robot; D/A—digital-to-analog converter; µC—microcontroller; PC—personal computer.

The view of the matrix transducer as well as a diagram of the measuring system are presented in Figure 1 and Figure 2. The matrix transducer has two subunits for excitation and pick-up function [12]. The excitation section (EXC) is built using a coil wound on a rod ferrite. Whole construction is centrally placed and generates an axially uniform field, which operates in the perpendicular direction to a surface of a tested material. Any disturbance of the field is sensed by eight digital magnetic sensors (*s*_1_–*s*_8_). The sensors are placed over the circumference of the transducer with a spacing of 45°. Each sensor allows for observation of the three-dimensional magnetic field vector *V* = (*V*_x_, *V*_y_, *V*_z_). The sensors are positioned so that the *x* sensitivity axis of each one is tangential to the circumference (Figure 1). Monitoring of all three components provides information that can be crucial for the identification of defects. Observation of the shape and the gradient of magnetic field disturbance over the examined object results in growth of the evaluation accuracy of not only depth, but also orientation or dimension of the defects [9,10,11,14,36]. The information content of the measured magnetic field can be analyzed under different terms providing various data about the defect parameters (Table 1).

Considering the frequency of the excitation waveform and the standard penetration depth, the material can be evaluated at different depths. In this paper, in order to obtain the highest discrimination between defects of different depths, the DC (direct current source) waveform was used. In a material having homogeneous electric and magnetic properties, each sensor is affected by the same field. Only in the case of some inhomogeneity arising near one or a group of sensors, would there be a difference noticed in the symmetry of the detected field. 

## 3. Defect Evaluation Procedure

**Figure 3:**
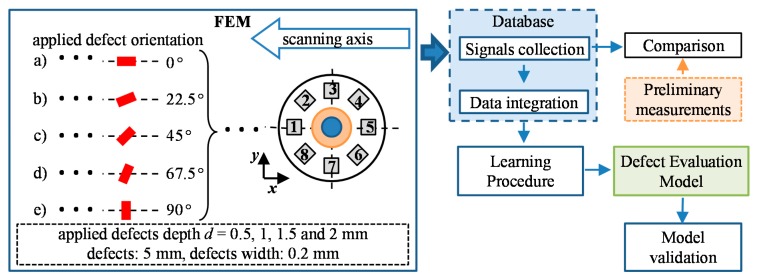
The schematic diagram of the definition of the defect characterization model; FEM—finite element method.

The diagram of the utilized defect evaluation procedure is presented in Figure 3. First, in order to prepare a database containing various possible configurations of the defect arising in the examined steel element, FEM numerical simulations were run using the COMSOL software. The defects having successively selected depths and orientation within the whole range of physical dimensions of the tested object were considered. The set of magnetic field vectors *V* sensed by the multi-sensor transducer was then collected. In order to verify the conditions applied during the computational process, the numerical simulation results were compared with the measurements for several selected defect arrangements. Next, in order to combine the data gathered by the matrix of sensors, the data integration algorithm based on fan-beam tomographic reconstruction procedure was utilized. Finally, three DCNN structures were constructed and used to carry out the defect characterization.

### 3.1. FEM Computations and Database Construction

**Figure 4:**
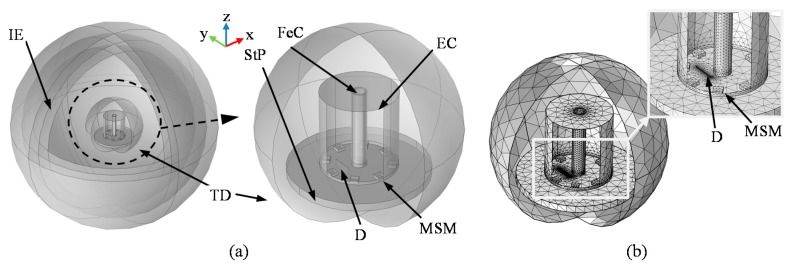
Utilized FEM model of the transducer: IE—infinite element domain, TD—transducer’s domain, StP—steel plate, MSM—multi-sensor matrix, FeC—ferrite core, EC—excitation coil, D—defect; (**a**) model view, (**b**) computation mesh view.

First, the universal FEM model geometry was built allowing automated reconfiguration of defect arrangement in the examined plate. The defect length and width were constant during the whole simulation process and equal to 5 mm and 0.2 mm, respectively. The view of the utilized model’s geometry is presented in Figure 4. During the initial stage of FEM modeling, the selection of simulation parameters was made in order to adjust the computation conditions to the one existing during the real measurements. For that reason, the physical parameters of real materials, such as steel sample or ferrite core, were used. In this paper, the computations were made under a DC excitation field, and the achieved field strength resulted in operation in the linear range of the ferrite core hysteresis. Additionally, the simulation parameters were also selected based on the compromise between the numerical error caused by the finite element dimension and computation time required for a single case analysis. The chosen density of utilized FEM mesh guaranteed having at least 10 elements within the smallest dimension in the vicinity of the examined plate and transducer while computation time of a single case was less than 10 minutes. Finally, the mesh consisted of around 5.4 ×10^5^ of tetrahedral elements. The objective of FEM numerical simulations was to provide the magnetic vector field distribution in the vicinity of the transducer for different orientation and position of defects in the material in reference to the transducer. The simulations were carried out for several different angles of the defect orientation with regards to the scanning axes (Figure 3) in the range between 0° (longer diameter of defect *l* was along the scanning direction) and 90° (*l* was across the scanning direction). Additionally, defects of different depths ranging from 0 mm (defect not present in the plate) to 2 mm (defect across the plate thickness) were also considered. In the successive step of the simulations cycle, the defect modeling domain element D (Figure 4) was shifted by a set distance in the *x* (constant and equal to 1mm) or *y* (adjusted to half of the distance in the *y* direction between the sensors, i.e., *s_1_* and *s_2_* or *s_2_* and *s_3_*) axis of the coordination plane (within the area of 29 × 10 mm; Figure 3). Finally, there were nearly 3.5 × 10^3^ 3D simulations processed. Despite the finite number of analyzed defect configurations, the computed cases supply a family of the characteristics of the transducer’s responses obtained for successive arrangements of the defect depths and orientation within the whole range of physical dimensions of the tested object. 

**Figure 5:**
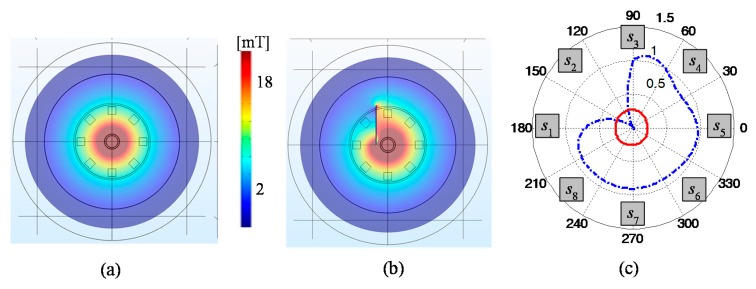
Selected results of flux distributions obtained during FEM simulations: (**a**) without defect; (**b**) with defect; (**c**) polar plot of the *V*_z_ component of the flux sensed by the successive sensors normalized to maximum value.

**Figure 6:**
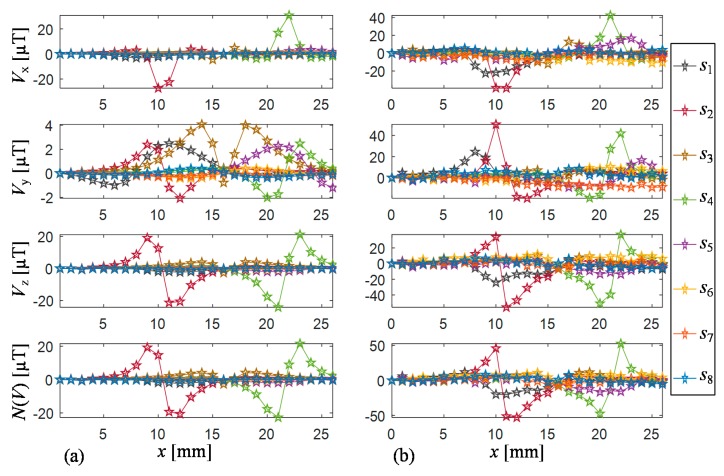
Comparison of selected results of magnetic field vector components acquired by all sensors for 1D scan along the 100% defect aligned at 0° to scanning direction obtained during FEM numerical simulations (**a**) and measurements (**b**).

Exemplary results of the simulations are shown in Figure 5 presenting the magnetic flux density distribution in the sensors’ matrix plane over the steel plate for two cases: without and with the defect of 0.5 mm depth in the examined sample. One can notice that the magnetic field circumferential distributions observed by the sensors allow relatively precise localization of the area where the defect occurs. After the computation process, the agreement of numerical characteristics with real measurements was evaluated. For that need, the 2D measurements were carried out for the same orientations of 100% defect as used during the FEM computations. Then, the extreme values and their positions as well as general characteristics were compared. Exemplary results achieved under selected defect arrangement are presented in Figure 6. The major difference between simulations and measurements can be observed in the case of corresponding component value levels. This can be explained by some differences in the physical parameters of the examined steel plate or ferrite core in comparison to the ones utilized during computations according to the data provided by producers. Additionally, there could be a slight difference in location of the collection (measuring) points in both cases. Nevertheless, it can be noticed that there is good agreement of the obtained signal distributions under corresponding conditions. The actual location, orientation (in respect to the transducer), and the depth of defect influence the obtained patterns. Similar behavior of the component’s value changes is visible in both cases. Therefore, just a standard normalization procedure was applied to the gathered signals in the database. Considering all possibilities of defects arising under the transducer’s sensitivity area, a number of patterns are introduced. For each single data collection point, the transducer provides a wide range of data collected by sensors, which are located over the circular orbit. Therefore, in order to combine the multi-sensor data and to obtain the general reconstruction corresponding to magnetic field response within the space covered by the transducer, the integration algorithm was applied [12]. The proposed procedure is based on the fan-beam tomographic reconstruction technique [37] and distinguishes the field distributions whenever nonuniformity occurs within the sensitivity range, regardless of the direction and location of its source (the details can be found in [12]). In order to calculate the projections, the magnetic vector norm value was utilized N(*V*). As a result, for each position of the transducer in reference to the defect (computation cell), a reconstruction image corresponding to the sensed field distribution was achieved. 

**Figure 7:**
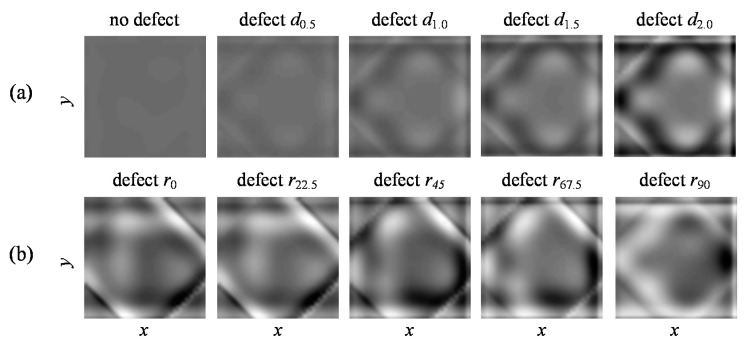
Selected results of reconstruction procedure obtained for: (**a**) different depth of the defects aligned at 0° (*r*_0_); (**b**) different orientation angle and depth of 2 mm (*d*_2.0_); the size of each reconstruction is 43 × 43.

The exemplary reconstruction images are presented in normalized scale (referred to the extreme values in database) in Figure 7. It is possible to see that the growth of the defect’s depth (Figure 7a) from 0.5 mm (*d*_0.5_) to 2 mm (*d*_2.0_) results in successive changes of the image gray level intensity range. In the reference, when there is no defect occurring within the sensitivity range of the transducer, the reconstructed image presents almost uniform color corresponding to the lower band of value ranges. Moreover, it is also possible to notice the changes of patterns in the images with the changing rotation *r* of the defect. Despite the clearly visible diversity in the obtained reconstructions, the definition of the relationship is not trivial and requires deep analysis to find hidden patterns. In such cases, as was discussed earlier, a deep learning procedure was found to be a very promising technique. Therefore, DCNN was utilized as a solution of the problem.

### 3.2. Multi-Label Classification

The DCNN is based on classical convolutional neural networks and is defined in a series of layers forming one or more hidden layers [18,20,22,23,28]. The deep multiple layer structure defines functions describing even complex problems simultaneously, resulting in higher efficiency and generalization capability. On the other hand, it requires a much broader database during the training process. The DCNN consists mostly of sets of three main components, which can be defined repeatedly on successive levels: convolutional, activation function, and subsampling layer [18,20,26]. The convolutional layer forms a set of weights between input and output creating filters realizing the convolution operation with the image by sliding over small sub-regions and resulting in a feature maps definition. Then, often a nonlinear transformation of data is carried out by applying an activation function such as a hyperbolic tangent function or rectified linear units (ReLU). In the subsampling layer, the information provided by a convolutional layer is combined by local pooling operations using maximum or average operators. Additionally, a fully connected layer (combining all of the features learned by hidden layers for larger patterns identification) and final classification layer are utilized. 

**Figure 8:**
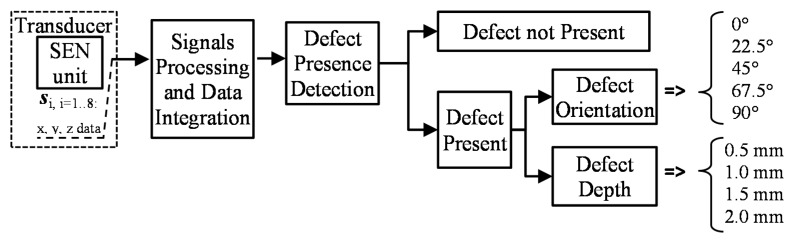
The block diagram of multi-class defect evaluation procedure from single point measurements.

**Figure 9:**
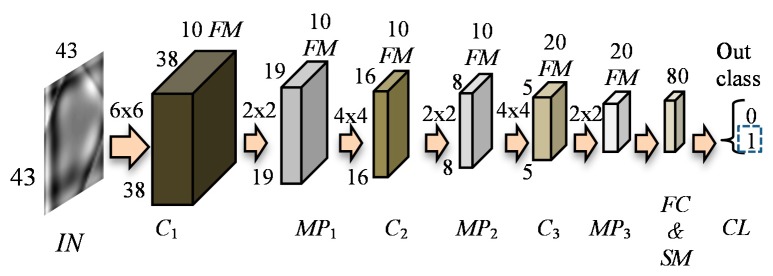
Schematic view of the deep convolutional neural network (*DCNN*_DND_) architecture for evaluation of defect occurrence; layers: *IN*—input, *C*—convolutional, *MP*—max-pooling, *FC* & *SM*—fully connected and softmax, *CL*—classification; *FM*—feature maps.

**Figure 10:**
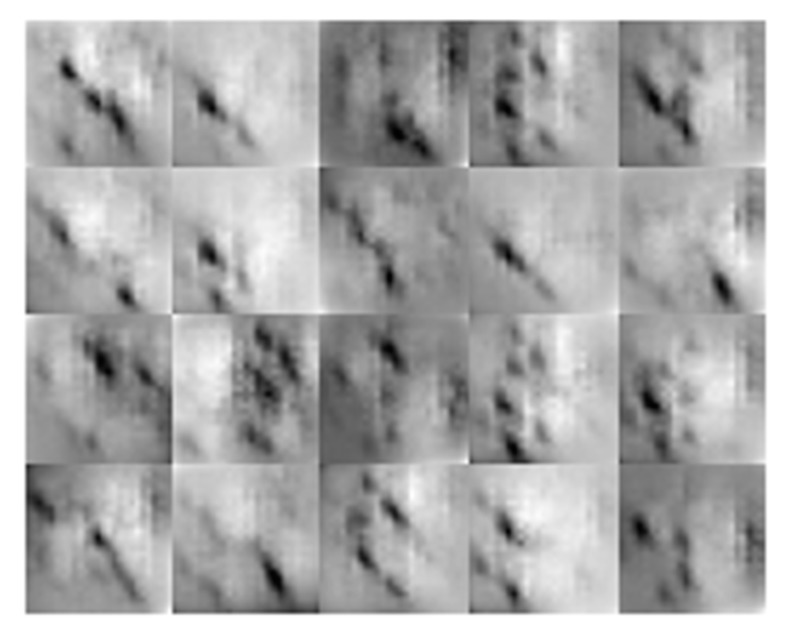
Visualization of the *DCNN*_DND_ network’s third convolutional layer response for random inputs.

In this paper, three different architectures allowing conduction of the multi-label classification were used for evaluation of defect occurrence (*DCNN*_DND_), rotation (*DCNN*_ROT_), and depth (*DCNN*_DEP_). Division of the defect evaluation process into separate stages allows utilization of the whole database for optimization of the structure and maximization of accuracy of each classification problem. The general diagram of the evaluation process is presented in Figure 8. First, for a single collection-position (computation cell) reconstructed-image, a defect occurrence is evaluated by running a classification network *DCNN*_DND_. Then, if the defect is estimated to be present, both depth and orientation are evaluated by *DCNN*_DEP_ and *DCNN*_ROT_. In order to optimize the network structures, an iteration procedure implementing each DCNN with various combinations was applied. First, the training process was carried out considering different numbers of hidden layers, their configuration, feature maps, and the sizes of filters. During the optimization process, the layers’ structures were constructed with different numbers of configurable subsets *sl* forming a final subset *SL* followed by fully-connected (*FC*) and softmax (*SM*) layers: {*SL*; *FC*; *SM*}. Each considered subset *sl* contained convolutional (*C*) and max-pooling (*MP*) layers of different sizes. The numbers of used *sl* subsets in the *SL* definition ranged from 1 to 3, so the structure *SL* was defined as: *SL* = {*sl*_1_}, *SL* = {*sl*_1_, *sl*_2_}, or *SL* = {*sl*_1_, *sl*_2_, *sl*_3_}. For each subset *sl,* several kernel sizes and feature map (*FM*) numbers of the convolutional and max-pooling layers were used. In the case of the convolutional layer, the kernels having a size of 3 × 3, 4 × 4, and 6 × 6 together with feature map numbers of 10, 20, 30, and 60 were utilized. Simultaneously, in case of max-pooling, the number of feature maps was equal to 10 and 20 and the kernels having the size of 2 × 2, stride of 2, without and with padding of 1 were used. Then, the network configurations allowing for achievement of the greatest accuracy for testing were chosen. The parameters of the *DCNN*_DND_ structure are presented in Figure 9 and Figure 10. It has eleven stages consisting of three *C*, three *MP* (subsampling) and one *FC* layer. The normalized reconstructed gray scale image of the 43 × 43 size is filtered by *C* layer with a 6 × 6 kernel and a number of 10 *FM*, and then twice with a 4 × 4 kernel having 10 and 20 *FM,* respectively, in the following stages. In each stage, *MP* operation was applied with the size of 2 × 2 and a stride of 2. As an activation function, a ReLU was used. Finally, the *FC* layer was utilized allowing further division into two classes (defect-free and defect). In the case of *DCNN*_ROT_, the input was followed by a *C* layer with 3 × 3 kernel and a number of 30 *FM*. Further, layers of: *FC* of the output size of 5, *SM* and classification (*CL*) follow the *MP* of a size 2 × 2, stride of 2, and padding of 1. In the case of *DCNN*_DEP_, a similar structure was used with the exception that the number of *FM* in *C* layer was 10, and there was no *MP* layer while the output size of *FC* layer was 4. In both cases, the cross channel normalization with four channels per element and ReLU as an activation function were utilized. For each classification problem, the database was analyzed separately. First, all cases of each considered class were divided into training and testing subsets according to the ratio of 85:15. Next, during the optimization process of DCNN structure, for each analyzed configuration, a five iterations cross-validation process was carried out on the training subset. Before training, the subset was shuffled once. During each round, the training was carried out on a randomly selected subset containing 85% of the cases. The remaining cases were used for validation. The evaluated efficiency of the structure was the average of obtained results. Considering the reduction of the computational time required for the whole optimization process, a mini-batch stochastic gradient decent approximation method was used during the training with an initial learning rate set to 10^−3^ and decay factor of 5 × 10^−4^ to avoid overfitting. The mini-batch size was set to 50 and maximum epochs number to 100. 

### 3.3. Verification of Defect Evaluation Model

For the purpose of the verification of the trained DCNN structures, the classification was run over the testing set. The confusion matrices of all three networks are presented in Table 2, Table 3 and Table 4. The rows correspond to the true classes and the columns to predicted ones of the testing sets. In the case of *DCNN*_DND_, the percentage of properly classified defects is at fairly good levels close to 92% and 82%. Analyzing the results achieved for *DCNN*_DEP_ and *DCNN*_ROT_, the worst level of classification accuracy was obtained for the middle classes with 58.97% for *r*_45_ and 54.35% for *d*_1.0_. The overall accuracy for *DCNN*_DND_*, DCNN*_ROT_, and *DCNN*_DEP_ was around 87%, 72%, and 70%, respectively. Additionally, for *DCNN*_DND_, the binary classification accuracy as well as the F1-score were calculated giving the results equal to 86.59% and 85.88%, respectively. The obtained accuracy rates should be analyzed in terms of magnetic circuits of the transducer and the way the electromagnetic field is generated and fed to the material. Considering the construction of the sensor, it should be pointed out that the sensitivity under the whole transducer’s area is not constant. The rate is mostly affected by misidentification of the states in regions close to the center of the transducer as well as close to its circumference. For some alignment of the defects located directly under the core (especially when they occurred centrally under the core; see Figure 6) the uniform distribution of flux is disturbed much more weakly by the defect (especially for shallower defects); in consequence, this can result in a lower rate of identification accuracy. In the second case, if the defect occurs close to the circumference of the transducer, the disturbance of the field arises close to the sensitivity area of the transducer. In consequence, it can affect only one or two sensors, which also results in a lower rate of identification accuracy. Therefore, even though the defect is located under the transducer’s area, it can be misclassified or even not detected. 

However, it should be stated that the evaluation is carried out separately each time for data reconstructed on the basis of signals gathered at a single given location, without the feedback providing the evaluation results from the surrounding area. For that reason, the achieved accuracy level of the obtained networks can be recognized as good. Nevertheless, in order to reach higher classification levels, the evaluation results achieved for neighboring areas can be used as a feedback of the classification procedure. Taking into consideration that the size of the evaluated defects is much greater than the step size of the transducer position, such procedures can reduce the influence of local insensitivity of the transducer.

### 3.4. Neighborhood Based Class Probability Update Algorithm

The concept of consideration of the neighboring cell state in the evaluation of the present state of a computation cell is frequently utilized in many applications dealing with spatial domains. To update the probability of a class assignment of a given cell, the cellular automata concept was adopted [38]. The model finds its application in problems described by arbitrary shaped cells arranged in grid-like structures. All cell state assignments are updated at the same discrete time steps according to predefined rules. The rule set is operating within the neighborhood of the cell under observation. 

**Figure 11:**
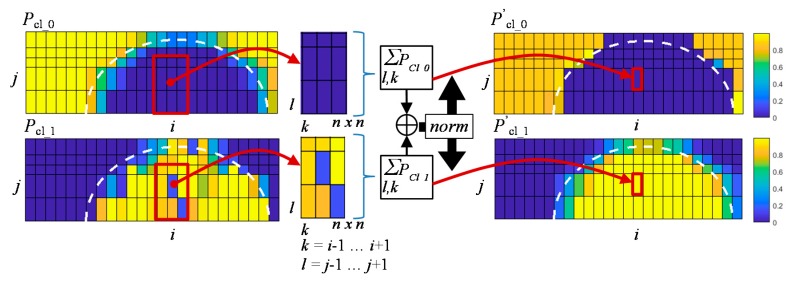
Visualization of the neighborhood based class probability update algorithm for the *DCNN*_DND_ case.

In this paper, a Moore neighborhood was utilized to calculate the new distribution of class probabilities [38]. The schematic visualization of the class probability assignment update algorithm for the *DCNN*_DND_ case is presented in Figure 11. First, the probabilities of both class occurrences (0—defect not present, 1—defect present within the sensitivity area of the transducer) over the whole considered area were predicted using the *DCNN*_DND_ model: where *P*_Cl_0_(*x*, *y*) is the probability of class 0 and *P*_Cl_1_(*x*, *y*) is the probability of class 1. Then, for each cell, the neighborhood mean probability of a given class assignment was calculated using the Moore neighborhood. The updated probabilities *P’*_Cl_0_(*x*, *y*) and *P’*_Cl_1_(*x*, *y*) in the observed cell were calculated utilizing the formula:

(6) $$\left\{ \begin{array}{l} P_{Cl\_0}^{\prime }(i,j)=\frac{\frac{1}{N}\sum_{k,l}P_{Cl\_0}}{\frac{1}{N}(\sum_{k,l}P_{Cl\_0}+\sum_{k,l}P_{Cl\_1})}\\ P_{Cl\_1}^{\prime }(i,j)=\frac{\frac{1}{N}\sum_{k,l}P_{Cl\_1}}{\frac{1}{N}(\sum_{k,l}P_{Cl\_0}+\sum_{k,l}P_{Cl\_1})} \end{array}\right.$$

where N is equal to 3 × 3, k = (*i* − 1,…,*i* + 1), and l = (*j* − 1, …, *j* + 1).

**Figure 12:**
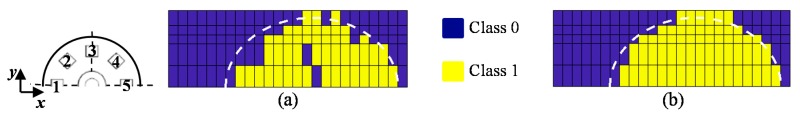
Visualization of the *DCNN*_DND_ class evaluation results: (**a**) before and (**b**) after utilization of class probability update algorithm; class 0—defect not sensed by the transducer, class 1—defect indicated by the transducer; white dashed line depicts the circumference of the transducer.

This procedure allows for the rewarding or penalizing of the class assignment confidence according to the generalized local neighborhood probabilities. Taking into consideration that the size of the defect is much smaller than the size of the cell, the algorithm can effectively increase the accuracy of the evaluation process. Finally, the class with the highest value of updated probability is assigned to the given cell. Exemplary results of prior and final 0-1 class assignment for the case of defect d(*r*_90_, *d*_0.5_) are presented in Figure 12. Similar procedures were defined and then carried out in the cases of depth and rotation evaluation results. 

In order to validate the performance of the algorithm, the classification was run once again over the testing set. The confusion matrices of all three networks are presented in Table 5, Table 6 and Table 7. It can be noticed that after the procedure, the proper classification rate is significantly higher. 

## 4. Conclusions

In this paper, a DCNN-based method allowing for discrimination between different states of the tested objects (also allowing evaluation of deeper layers of the material, not visibly accessed) using complex patterns of matrix multi-sensor transducer signals was presented. First, in order to obtain a database, finite element method numerical simulations were run, resulting in nearly 3.5 × 10^3^ cases. Despite the finite number of analyzed defects configurations, the computed cases obtained the family of transducer’s response characteristics for successive arrangements of defects’ depths and orientation within the whole range of physical dimensions of the tested object. The correctness of the simulation conditions was verified based on the preliminary measurements. Then, the data integration algorithm was utilized in order to achieve reconstructions of magnetic field distributions for different locations and orientations of defected areas under the transducer. The interpretation of the reconstructed data is not explicit (as for e.g., in the case of the visual inspection data) and, thus, requires a machine learning attempt. Therefore, to automate the process of feature extraction and definition of complicated characteristics for learning, the deep convolutional neural network DCNN was used. Three different network structures for defect occurrence, depth, and rotation assessments were optimized. The evaluated accuracies of the achieved networks reached satisfactory classification levels. However, the local evaluation results were obtained considering only the data form the analyzed computation cell without the information from the surrounding area. Therefore, an updated procedure for defect characterization was then proposed based on a cellular automata algorithm. The validation of the updated results confirmed significant improvement of the evaluation accuracy. However, the achieved high performance quality should be assessed only in terms of additional local information data fusion. The quality should be further evaluated for more complicated cases of defect arrangement.

Additionally, in future work, the accuracy can be increased by extension of the training database, especially for the classification of depth and rotation problems. The approximation procedure can be carried out for the obtained 3D FEM characteristics. As a result, it is possible to achieve the values for any configuration of defect arrangement between the simulated ones. Moreover, the analysis for combining the database from two modes (simulations and measurements) will be also carried out. Furthermore, in the case of rotation and depth evaluations, the regression DCNN structure will be optimized. This all will characterize the defect in higher ranges and assess the rotation or depth with higher accuracy.

The original construction of the multi-axis transducer obtains similar sensitivity for any nonuniformity affecting changes of magnetic properties which occur at any angle to the transducer x-y axis. Therefore, in future work, the transducer could be utilized to implement any application concerning characterization and mapping of damaged areas, their size (length, width, and depth), and orientation such as plastic deformations, fatigue changes, and stress concentration. Moreover, it could be applied to the directional inspection (e.g., anisotropy examination) of changes of mechanical properties under different technological or production processes such as inspection of case hardening.

## Figures and Tables

**Table 1:** Magnetic vector field expression modes.

Description	Definition	
Magnetic field norm (magnitude)	$N(V)=\sqrt{V_{x}^{2}+V_{y}^{2}+V_{z}^{2}}$	(1)
Magnetic field tangential component	$V_{t}=\sqrt{V_{x}^{2}+V_{y}^{2}}$	(2)
Magnetic field normal component	$V_{n}=V_{z}$	(3)
Angle between the normal and tangential components	$\phi (V)=\arctan(V_{n}/V_{t})$	(4)
Angle between the tangential components	$\phi_{t}(V)=\arctan(V_{x}/V_{y})$	(5)

**Table 2:** Confusion matrix of the *DCNN*_DND_ [%].

	Defect-free	Defect
Defect-free	91.57	8.43
Defect	18.40	81.60

**Table 3:** Confusion matrix of the *DCNN*_ROT_ (rotation) [%].

	Defect *r*_0_	Defect *r*_22.5_	Defect *r*_45_	Defect *r*_67.5_	Defect *r*_90_
Defect *r*_0_	74.36	7.69	10.26	2.56	5.13
Defect *r*_22.5_	12.82	79.49	7.69	0	0
Defect *r*_45_	5.13	12.82	58.97	5.13	17.95
Defect *r*_67.5_	0	0	2.56	71.80	25.64
Defect *r*_90_	0	2.56	0	20.51	76.93

**Table 4:** Confusion matrix of the *DCNN*_DEP_ (depth) [%].

	Defect *d*_0.5_	Defect *d*_1.0_	Defect *d*_1.5_	Defect *d*_2.0_
Defect *d*_0.5_	82.61	10.87	6.52	0
Defect *d*_1.0_	21.74	54.35	19.56	4.35
Defect *d*_1.5_	2.17	19.57	67.39	10.87
Defect *d*_2.0_	6.52	6.53	13.04	73.91

**Table 5:** Confusion matrix of the *DCNN*_DND_ after evaluation update procedure [%].

	Defect-free	Defect
Defect-free	95.12	4.88
Defect	5.99	94.01

**Table 6:** Confusion matrix of the *DCNN*_ROT_ after evaluation update procedure [%].

	Defect *r*_0_	Defect *r*_22.5_	Defect *r*_45_	Defect *r*_67.5_	Defect *r*_90_
Defect *r*_0_	100	0	0	0	0
Defect *r*_22.5_	0	100	0	0	0
Defect *r*_45_	0	0	92.31	5.13	2.56
Defect *r*_67.5_	0	0	2.56	97.44	0
Defect *r*_90_	0	0	0	0	100

**Table 7:** Confusion matrix of the *DCNN*_DEP_ after evaluation update procedure [%].

	Defect *d*_0.5_	Defect *d*_1.0_	Defect *d*_1.5_	Defect *d*_2.0_
Defect *d*_0.5_	95.65	2.18	2.17	0
Defect *d*_1.0_	2.17	93.48	4.35	0
Defect *d*_1.5_	0	6.52	93.48	0
Defect *d*_2.0_	6.52	0	4.35	89.13
